# How to bring residents’ psychosocial well-being to the heart of the fight against Covid-19 in Belgian nursing homes—A qualitative study

**DOI:** 10.1371/journal.pone.0249098

**Published:** 2021-03-26

**Authors:** Sanne Kaelen, Wilma van den Boogaard, Umberto Pellecchia, Sofie Spiers, Caroline De Cramer, Gwennin Demaegd, Edouard Fouqueray, Rafael Van den Bergh, Stephanie Goublomme, Tom Decroo, Muriel Quinet, Elke Van Hoof, Bertrand Draguez

**Affiliations:** 1 Belgium Covid-19 Project, Médecins Sans Frontières, Operational Centre Brussels, Brussels, Belgium; 2 Médecins Sans Frontières, Operational Centre Brussels, Operational Research Unit (LuxOR), Luxembourg, Luxembourg/Brussels, Belgium; 3 Institute of Tropical Medicine, Antwerp, Belgium; 4 Iriscare, Public Health Institute, Brussels, Belgium; 5 Working Group on the Psychosocial Impact of the COVID-19 Pandemic Within the Superior Health Council, Brussels, Belgium; 6 Mental Health Sub-working Group, GEES, Brussels, Belgium; 7 Vrije Universiteit Brussel, Brussels, Belgium; Iwate Medical University, JAPAN

## Abstract

**Background:**

Nursing homes (NH) for the elderly have been particularly affected by the Covid-19 pandemic mainly due to their hosted vulnerable populations and poor outbreak preparedness. In Belgium, the medical humanitarian organization Médecins Sans Frontières (MSF) implemented a support project for NH including training on infection prevention and control (IPC), (re)-organization of care, and psychosocial support for NH staff. As psychosocial and mental health needs of NH residents in times of Covid-19 are poorly understood and addressed, this study aimed to better understand these needs and how staff could respond accordingly.

**Methods:**

A qualitative study adopting thematic content analysis. Eight focus group discussions with direct caring staff and 56 in-depth interviews with residents were conducted in eight purposively and conveniently selected NHs in Brussels, Belgium, June 2020.

**Results:**

NH residents experienced losses of freedom, social life, autonomy, and recreational activities that deprived them of their basic psychological needs. This had a massive impact on their mental well-being expressed in feeling depressed, anxious, and frustrated as well as decreased meaning and quality of life. Staff felt unprepared for the challenges posed by the pandemic; lacking guidelines, personal protective equipment and clarity around organization of care. They were confronted with professional and ethical dilemmas, feeling ‘trapped’ between IPC and the residents’ wellbeing. They witnessed the detrimental effects of the measures imposed on their residents.

**Conclusion:**

This study revealed the insights of residents’ and NH staff at the height of the early Covid-19 pandemic. Clearer outbreak plans, including psychosocial support, could have prevented the aggravated mental health conditions of both residents and staff. A holistic approach is needed in NHs in which tailor-made essential restrictive IPC measures are combined with psychosocial support measures to reduce the impact on residents’ mental health impact and to enhance their quality of life.

## Introduction

The Coronavirus Disease-2019 (Covid-19), has ravaged Europe and most of the world since early 2020. It has disproportionately affected the elderly, particularly those living in close and continuous proximity in nursing homes (NH) worldwide [1, 2].

Covid-19 spreads rapidly in the absence of appropriate infection prevention and control (IPC) measures, and carries a high risk of severe morbidity and mortality among the elderly [3]. The alarming number of Covid-19 infections and deaths recorded in NHs has been attributed to a lack of protocols for implementing IPC measures, lack of training and support for NH staff, and insufficient personal protective and medical equipment for staff and residents [4]. In Belgium, at the end of the first wave, late June 2020, over 6200 NH residents had died, accounting for more than half of confirmed Covid-19 deaths in the country [5].

In close collaboration with Belgian local health authorities, the medical humanitarian organization Médecins Sans Frontières (MSF) supported more than 130 NHs across Belgium during the first wave, from mid-March to the end of June 2020, to implement IPC measures, reorganize care, and provide psychosocial support for staff. Late in May 2020, MSF conducted an online survey in 983 NHs all over Belgium. Nine out of ten NHs reported exacerbated or new psychological symptoms such as sadness, depressive symptoms and deterioration of cognitive abilities amongst residents due to quarantining and the stringent lockdown measures [6], which in Belgium essentially entailed that NH were closed from one day to the other. These measures created a general condition of loneliness according to Roy J. et al. [7] Many studies show the detrimental effects of social isolation and loneliness due to quarantine on mental health [8–10], including descriptions of the impact on psychosocial health during the SARS outbreak in 2004 [11–13]. Currently, several researchers have warned about the psychological impact of quarantine and social isolation of the elderly during the Covid-19 pandemic [14–16].

Psychosocial support to residents of NHs is challenging at the best of times [17], although different models to provide it do exist [18, 19], often relying on external actors [20]. During Covid-19, these problems were compounded: mental health distress among residents was exacerbated due to loneliness and uncertainty, external support was no longer possible, and staff themselves were scared and traumatised. Only very limited literature exists on this particular combination of circumstances. Furthermore, according to our knowledge, no study has been conducted during the first Covid-19 wave on how residents perceive and experience the outbreak prevention measures being taken for them by the NHs. We therefore conducted a study to understand in depth residents’ mental health needs during this “perfect storm”, and to identify processes or structures through which NH staff could be supported to better address those needs. The ultimate goal was to contribute to policies that better address mental health issues associated with the Covid-19 lockdown.

Therefore, the aim of this study was to explore and better understand: 1. which psychosocial and mental health needs were identified and experienced by residents of NHs during the first wave of the Covid-19 pandemic, and 2. how NH staff perceived and experienced their preparedness for addressing those needs.

## Methods

### Design

This was a qualitative study adopting a thematic content analysis. Qualitative data was collected by conducting focus group discussions with direct care staff and 56 in-depth interviews with residents in eight purposively and conveniently selected NHs in Brussels, Belgium, June 2020.

### Study population

Four NHs were purposively sampled from those where MSF had already intervened with support activities. The status as private, public, or non-profit facility was considered in this selection. Another four were selected through convenience sampling and were visited for the first time by MSF in the context of this study. From a list of all NHs in Brussels where MSF had not intervened, a NH was identified randomly and asked for willingness to participate, until four NHs were matched.

The study population consisted of NH residents who were able to understand the purpose of the study and to provide a conscious informed consent were included. The MSF psychologist, who was also the principal investigator, conducted this pre-selection using the mini-mental state examination tool. This tool provides a score up to 30 points and in which a score below 24 points suggests a level of dementia. Only those residents scoring between 24–30 points were eligible participants [21]. Further criteria taken into account were the diversity in the degree of social connectedness before Covid-19 as this could potentially influence their mental wellbeing and need for psychosocial support. All participants needed to be able to communicate in Dutch and/or French.

Out of the 64 selected resident participants, eight residents declined to participate at the appointment date and time. Reasons for declining were fatigue, illness, other appointments, or having lost the interest to participate. Among the remaining 56 participating residents, 35 were female and 21 were male. The median age was 85, ranging from 58 to 101 years (Table 1).

**Table 1:** Overview of residents’ characteristics in eight nursing homes in Brussels, Belgium, June 2020.

Age	< 60		60–69		70–79		80–89		> 90		TOTAL	
Gender	*F*	*M*	*F*	*M*	*F*	*M*	*F*	*M*	*F*	*M*	*F*	*M*
**NH1**			1		2		2	1			5	1
**NH2**						1	2	2	2		4	3
**NH3**				1		2	2	1	2		4	4
**NH4**					1		1		3	2	5	2
**NH5**					1	1	3	1		1	4	3
**NH6**	1				1	3				1	2	4
**NH7**					1		5		1	1	7	1
**NH8**					2	1		1	2	1	4	3
**TOTAL**	1		1	1	8	8	15	6	10	6	35	21

F, female; M, male; NH, nursing home

The second study participants’ group were the staff who were in direct contact with the residents from the eight identified NHs during the Covid-19 lockdown period. In the sampling procedure of participating staff, we aimed to reach sufficient variation among staff disciplines. Criteria were a mix of medical and support staff who did not have any hierarchal relation to each other. Each focus group discussion had three to seven participants, and a total of 44 staff-members were included. The majority, 38 out of 44, was female and most were nursing aid assistants. The working experience in their current NH was on average 10 years (Table 2).

**Table 2:** Overview of direct caring staff job profiles in eight nursing homes in Brussels, Belgium, June 2020.

	Nurse	Nurse-aid	Occupational or physical therapist	Support staff ¥	TOTAL
**NH1**	1	1	1		3
**NH2**	1	5			6
**NH3**	1	3	1	1	6
**NH4**	2	2	1	1	6
**NH5**	1	1	2	3	7
**NH6**	1	2	2		5
**NH7**	2		2	2	6
**NH8**	1	3		1	5
**TOTAL**	10	17	9	8	44

**¥** Support staff: kitchen staff, cleaning staff, administrative profiles

### Data collection

The data was collected through in-depth interviews with residents and focus group discussions with staff from June 15^th^ to July 3rd 2020, conforming with Green & Thorogood’s qualitative methods for health research [22]. Two experienced qualitative researchers (UP/WvdB) trained and coached five bilingual (Dutch and French) project staff members (CDC/GD/EF/SK/SS) prior and during the data collection.

The in-depth interviews and focus group discussions were guided by a topic list that was further elaborated into question guides with prompts (Table 3). All questions concerned the period of the first wave in Belgium, from March to the time of the interview. These question guides were pre-tested in a NH where MSF had intervened before and where NH staff felt comfortable with MSF staff. This NH was not part of the study and served besides the pre-testing as a training ground for the data collection team.

**Table 3:** Topic guides for in-depth interviews with residents and focus group discussions with staff in eight nursing homes in Brussels, Belgium, June 2020.

In-depth interviews with residents	Focus group discussions with staff
• Knowledge of Covid-19 pandemic • Understanding of the pandemic impact • Source(s) of information and type of communication about the pandemic • Source(s) of information and type of communication about how the Covid-19 can affect their residence • Knowledge of facility’s measure to prevent the epidemic • How the Covid-19 measure impacted in their lives / thoughts / daily activities / interaction with other residents / interaction with social network • Emotional impact of the epidemic and measures • Experience around zoning and cohorting • Experience around staff behaviours • Experience around other residents’ behaviours • Meaning and impact of isolation • Perceptions of loneliness / fear / risks • Needs satisfied / unsatisfied during the period of the epidemic • Perception of the reasons why the epidemic impacted in that specific way in their residence • Opinion on what went well / not well in the specific way the epidemic was managed	• Knowledge about the in-house outbreak preparedness plan, including psychosocial interventions and MH assessments • Contents of the outbreak plan, if existing • Level of preparedness of the structure, with emphasis to psychosocial aspects Acceptability and perceived appropriateness of the outbreak preparedness plan • What is perceived to be lacking/could be done differently in relation to how they were comfortable with operationalizing the plan and if and how psychosocial needs changed among the recipient residents • Reasons why preparedness did not work / worked partially / worked successfully • Practices put in place to respond to the needs of the residents in case of absence of a structured plan • Needs of the residents • Barriers encountered in residents’ care, with emphasis on wellbeing • Perception of the impact of isolation • Needs of the nursing staff • Experiences of collaboration with other professionals: GPs, psychologists

Most in-depth interviews took place in the residents’ own rooms. In some cases, this was not allowed, due to strict IPC measures of the NH, in which case in-depth interviews were conducted outside in the garden or inside in a big open space where privacy and social distancing could be respected. The in-depth interviews lasted approximately 45–60 minutes.

The focus group discussions took place in a private and/or comfortable site in the NH chosen by the participating staff and lasted around 90 minutes.

Psychological support was made available, in-house or by MSF, for all respondents during and/or after the in-depth interviews or focus group discussions.

All in-depth interviews and focus group discussions were audio-recorded and were then de-identified and either transcribed verbatim into French or transcribed into English if the interview was conducted in Dutch. Only de-identified transcripts were used by the principal investigator and two other study team members for analysis.

### Data analysis

All de-identified transcripts were analyzed manually by the principal investigator and two co-investigators. Codes and themes were generated using thematic content analysis as described by Green and Thorogood [22]. At the first stage, the transcripts were re-read and codes were ascribed to text sections. To ensure reliability of the coding procedure, the first interviews were independently coded by SK and UP, which revealed high consensus. Continuation of the analyses was done by SK, UP and WvdB. The same three researchers discussed the various codes and jointly identified patterns that were developed into main themes. These then were discussed until consensus was reached. Triangulation took place by comparing the residents’ responses with the staff responses on the same codes/themes.

### Ethics approval

This study was approved by the MSF Ethics Review Board, Geneva, Switzerland and the Institutional Review Board of the Institute of Tropical Medicine, Antwerp, Belgium. The management teams in the nursing homes gave their approval for the research to be carried out in their specific NH. Written informed consent to participate in the study, to publish the results and to use (anonymized) quotes was obtained from all respondents. All respondents were able to withdraw from the study at any point in time.

## Results

The following section describes the living experiences and perceptions of NH residents and staff through an analysis of the themes emerging (Tables 4 and 5) from the narratives of the in-depth interviews and focus group discussions.

**Table 4:** Emerging themes from the narratives of residents, with codes and sub-codes in eight nursing homes in Brussels, Belgium, June 2020.

Residents	
*Codes and Sub-codes*	*Theme*
Experience and perception of prohibition of movements;	Loss of freedom
Experience and perception of isolation from outside world;	
Forced changes in habits (shopping, laundry);	
Prison metaphor;	
War metaphor;	
Experience of eating alone;	Loss of social life
Experience and perception of blockage of entertainment activities;	
Absence of family visits;	
Absence of friends’ visits;	
No more contact with other residents;	
Fear of loneliness	
Boredom;	Loss of distraction and stimulation
Absence of distraction;	
Oversleeping;	
No more physical therapy;	
Losing interest in doing anything	
Source of information about the pandemic;	Loss of autonomy
Access to information (media; TV; newspapers);	
Knowledge about the containment measures;	
Feeling abandoned;	
Feeling treated as a child;	
Feeling useless / unworthy;	
Not asked for suggestions;	
Excluded from decision-making process	
Lockdown: anger, stress, depressive feelings;	Perception of own wellbeing
Losing hopes, feeling negative;	
Losing interest in life;	
Being together with other residents;	Needs
Family visits;	
Being treated respectfully;	
Activities, distractions;	
Fair information;	
Human contact;	

**Table 5:** Emerging themes from the narratives of nursing home staff, with codes and sub-codes in eight nursing homes in Brussels, Belgium, June 2020.

Nursing home staff	
*Codes and Sub-codes*	*Theme*
Use of masks;	Incoherent information and communication
Lack of meetings and formal info sharing	
Lack of masks and tests;	Lack of personal protective equipment and test
Changing plans;	Re-organization of work
Sudden tasks shifting;	
Informal meetings / peers decision making;	
More workload;	
Use of Skype for residents;	
Loss of human contacts;	Emotional effects
Ethical dilemmas;	
Sense of sadness for residents;	
Sense of being abandoned as professionals;	
Stress;	
Commitment;	
Attachment to residents	
Coherent organization;	Needs
Guidelines;	
Allowing family visits	

### The consequences of lockdown measures on residents’ lives

Loss of freedom

*“More than my health*, *I really feel that it is freedom that I have been robbed of and I find it difficult to bear*.” *(NH5*, *73 years*, *male resident)*

As a resident said, “*it is horrible to be locked up*” and the recurrently used metaphor of “*being in a prison*” illustrated the sense of having lost their freedom. It portrayed the notion of being separated from the outside world, where life goes on while theirs is put on hold. The residents’ daily routine became increasingly threatened due to the restrictive measures in the NH. Their daily habits and rhythms changed, for example, as they could not go out for personal shopping or hobbies.

With the exception of one NH, all residents were temporarily isolated in their rooms. Residents reported feeling *“claustrophobic”*, *“becoming crazy”*, or *“depressed”*. They reported that the experience of being in lockdown was “*the most horrible period of their life*” even compared to the period of World War II.

*"So the difference [between war and confinement] is that confinement was never there*. *[*…*] people always had the freedom to go out*, *to go out freely*. *If you went out or if you stayed at home*… *there were no instructions*.” *(NH3*, *94 years*, *female resident)*

#### Loss of social life

To protect NH residents and staff against the virus, contact with persons outside the NH was no longer allowed; residents could not see their family and friends. For some this even meant not seeing their own partner. Besides family and friends, professional primary care providers such as general practitioners and psychologists were no longer entering the NHs.

*“[*…*] All right*, *we were locked up*, *we could not get out*. *We have nothing*, *nothing*. *And to finish this*, *my wife is unhappy over there and I am unhappy here too*.*” (NH2*, *87 years*, *male resident)*

An additional aspect of losing social life was the social disconnection inside the NHs. Not uncommonly, residents were not allowed to interact with other residents; hairdressers and other support services were stopped and the last social resort, the direct caring staff also had to remain distant.

“*They [the staff] had no time anymore*. *They had to be very fast*, *and they disappeared quickly*. *For example*, *when they bring lunch*, *they put the tray down and leave*. *And as my mind became a bit slow*, *I think about all those questions I want to ask when they have already left the room*.*” (NH1*, *79 year*, *female resident)*

This meant in practice that there was an abrupt stop to any social and physical contact with either staff or other residents, creating widespread feelings of solitude and loneliness. For some, it felt as if they had been *“abandoned”*. They described that the entire atmosphere had changed within the NH: it became “*cold’*, “*distant*”, and there was “*no more joy*”.

“*We are social beings*. *One is not made to stay alone in one’s room*, *it is enough to become crazy*. *We can read*, *we can watch television*, *but it is not the same thing as living with others*.*” (NH5*, *80 years*, *female resident)*

“*The lack of contact*, *I didn’t hold anybody since… I think the lack of physical contact is very hard*. *It’s almost unhuman*. *It’s not natural*.*” (NH7*, *76 years*, *female resident)*

#### Loss of distraction and stimulation

All recreational activities were cancelled, creating a great sense of boredom, a lack of distraction and stimulation. Only adding more to the loss of social life mentioned earlier on.

“*And when you are very old*, *all of this (activities) stimulates you strongly*. *Basically*, *there is no stimulation anymore*, *only what you create yourself*, *going for a walk and all that*, *if not*, *there is nothing left*. *There was nothing anymore*.*” (NH7*, *87 years*, *female resident)*

The lack of primary health care and other support services created an impoverished environment, as those services were also considered essential distractions.

“*The physiotherapist*, *the hairdresser*, *the pedicure*, *they couldn’t come and we missed this*. *For us it is a way of distraction as well*. *Because for example the pedicure*, *she told us a bit about her son*, *and it distracts us*. *And the hairdresser*, *you know how that goes*, *they talk as well*, *they ask some questions*. *And with the physiotherapist I do my exercises*. *In the end we have pain everywhere*, *because of staying inactive*, *we miss it*, *for 3 months 3 and a half months*, *nothing*.*” (NH1*, *88 years*, *female resident)*

#### Loss of autonomy

There was a real absence of proactive communication and information sharing from the NH staff to the residents, except for one NH which due to its small size had already participatory information sharing in place. Residents were informed about the virus and general protective measures via various media channels but information about the Covid-19 situation in their own NH was not communicated; nor were the IPC measures explained to them. Sometimes residents had to receive this news via their own children, or other outsiders, which made them feel infantilized.

"*It is this that I cannot stand*, *it is unbearable*. *They [the staff] take you for pawns*, *for children*, *you see*. *They do not ask your opinion*, *and it is worse during the Covid [*…*]*. *We are very easily infantilized*.*" (NH5*, *80 years*, *female resident)*

The non or partially-received information and rules implemented by the NH staff resulted in residents often feeling unrecognized, forgotten, and deprived of the opportunity to share their struggles and concerns: “*It doesn’t matter what you as a resident say*, *they don’t even hear it*.*” (NH3*, *66 years*, *male resident)* Others were just mainly frustrated: “*The communication here sucks*, *but completely*. *You don’t have any information*, *not even about the virus*. *They don’t tell you anything at all but they informed my daughter*, *or other people from the family*, *but you*… *who are you*? *Nothing*.*” (NH5*, *83 years*, *male resident)*

Besides not being informed, residents were neither included in any decision-making processes, nor were they asked for their opinion. *“Our input was never asked*.*” (NH4*, *92 years*, *female resident)* There was no platform where they could ask their questions, raise their concerns or propose any suggestions that would have given the feeling to be part of the decision-makings, even unpopular ones. The not being informed, being infantilized, and being excluded from any decision-making made them feel losing their autonomy.

"*It’s a bit the feeling I have*, *in nursing homes are all old people*, *period*. *Handicapped people unable to decide for themselves*. *Trust the adult residents in this house for God’s sake*.*” (NH3*, *66 years*, *male resident)*

All the aforementioned ‘losses’ led the residents to consider that the lockdown measures were “*too long*”, *“unfair”* and *“illogical”* as staff were allowed to go in and out, but residents were not. During the reduced confinement phase this difference became only bigger; the general population in Belgium was allowed to go outside, but the residents from NHs weren’t. *“We are at the end of the list to restart a normal life*.*” (NH6*, *75 years*, *male resident)*

There were residents who said the measures were *“exaggerated”*, and others who pointed out that the diversity in a NH was not taken into consideration.

“*I personally think it’s an exaggeration*. *Yes*, *I know that in other homes 50 people have died*, *but aren’t they happier*, *do you think it’s fun to live here*? *They overprotect us*, *that’s not what we want*. *What we want is to be free*.*” (NH5*, *73 years*, *male resident)*

### Perceptions of residents’ wellbeing

Most residents had a resilient and accepting attitude at the beginning of the lockdown. But as the measures continued, they became increasingly angry and stressed. The measures implemented to curtail the spread of the Covid-19 took away their freedom and they had no perspective on when they would be able to see their family and friends again. This lack of perspective was accompanied by a deep existential uncertainty. It took away hope and trust in the future. To express their discomfort, respondents used words like: *“I am at the end of my rope*.*” (NH2*, *87 years*, *male resident)*. Over time, residents described they experienced more depressive symptoms. Gradually their feelings became heavier, more hopeless.

“*It was going well I can say for 2 months*, *I held out*. *But a while ago it changed*, *now the morale is at zero*. *But completely flat*. *I don’t feel like doing anything anymore*, *I have to force myself to do something to forget that I’m locked up but it’s not right*. *[…] You change*. *But it’s very subtle*, *I can’t explain how you change*. *But I don’t feel anymore like I did at the beginning*.*” (NH3*, *84 years*, *female resident)*

Residents reported feeling sad, losing interest in doing anything, having a low morale, losing hope, feeling negative, unworthy and useless. In some interviews we heard that the combination of powerlessness and solitude resulted in suicidal thoughts.

“*How am I doing*? *How do you want it to go*? *It can’t go anymore*. . . . *We don’t have a life anymore since Covid-19*. *Yes*, *I think more and more about*. . . . *suicide*, *because I think I’m at the end of a depression if it continues*. *Before I go to sleep*, *I wish I wouldn’t wake up the next day*.*” (NH6*, *70 years*, *male resident)*

Several residents reported cognitive and physical degradation. They experienced more tiredness, loss of appetite, and a loss of mobility. Different residents said they felt their memory was letting them down.

"*It deteriorates me a little bit*, *as if I start ‘losing my mind’*, *forgetting words and so on*.*” (NH1*, *77 years*, *female resident)*

However, there were also respondents who were able to face the situation, at least to a certain extent. They had somehow ‘chosen’ to give up resistance and tried to make life as bearable as possible.

Others said it was not so much a choice, but rather an attitude, a character trait.

“*With age*, *one relativizes more*.*” (NH4*, *92 years*, *male resident)*

Strikingly the notion of fear expressed by the residents was not related to being afraid of becoming infected by Covid-19 as nearly all residents said they were already at the end of their lives. Their expressed fear however was in relation to the next wave, for being locked-up and isolated again, and fear for dying alone.

"*No*, *I am not afraid [of Covid-19]*. . . . *I’m afraid it’s going to happen again and we have to go in quarantine again*. . . . *(NH4*, *79 years*, *female resident)*

“*A second lockdown will be horrible*, *I don’t want to think about that*, *because you know*, *I may die from one day to the next*, *so I prefer to be free*.*” (NH2*, *88 years*, *female resident)*

### Preparedness of nursing homes staff

#### Incoherent information and communication

For the staff, the beginning of the Covid-19 crisis was marked by unclear information and instructions on what to do and not to do in order to avoid contamination. NH staff reported they had no clear plan, there were no written guidelines. A respondent said *“I think what we missed is something very clear*. *It is a directive*, *if this happens you do this*, *and if that happens you do that*. *I think that is what we missed in the end*.*” (FGD NH1*, *Occupational therapist)* Plans were changing often, and contained a lot of incoherence. Formal meetings were cancelled at a time where communication was of utmost importance. All respondents were particularly distressed by the lack of information.

"*We were faced with a situation we could not control*. *It is the unknown that frightens everyone*, *starting with the direction*. *Everybody*, *we were all lost*. *As there was information being added each time we were all lost*, *we did not know what to focus on” (NH6*, *Nurse-aid)*

#### Lack of personal protective equipment and testing

Beside the lack of information, NH faced a huge lack of essential supplies. The shortage of personal protective equipment (PPE) left most staff-members without adequate or even any protection for weeks.

“*In isolation it was an outfit for all the staff*. *That is to say*, *you go to “x” with the same gown*, *you go out and you have to go to “y” with the same gown*, *and with the same mask*. *(NH2*, *Nurse)*

According to the staff there was no regular testing performed in their NH; the tests were not made available for these institutions. This contributed to insecurity and anxiety on how to provide care for the residents, contributing and increasing the staff’s perception of risk of being infected.

“*But we did not have tests*, *so we had to isolate them*, *we did not know if they were positive or not*, *it was the only way to protect the others”*. *(NH5*, *Physiotherapist)*

#### Re-organization of work

Due to all the necessary IPC measures put in place, the workload of the staff increased substantially. From one moment to another, NH staff were given new roles. Some staff had to learn new tasks on the spot, re-organize their way of working and caring. This was true, particularly, for physiotherapists and occupational therapists as their tasks changed completely because they were no longer allowed to carry out their own jobs. Working in the kitchen or helping nursing staff with other caring tasks made them feel lost. *“For me in any case*, *in relation to my work*, *which is doing activities in the end*, *and there are no more*. *So*, *I was a bit lost*, *what am I going to do*?*” (NH3*, *Occupational therapist)*

In addition, as residents no longer had contact with their family members, staff felt that they had to replace the emotional support residents normally received from their families. Staff felt that they could no longer answer all the needs of the residents.

"*We were really torn between their happiness*, *their protection*, *and at the same time having to be a bit stricter and forcing them to stay in their rooms*. *It’s a bit abusive to tell someone we’re going to have to lock you up if you leave your room*, *for your safety*, *it doesn’t work for us*, *and in some cases we’ve been forced to lock them up if they’re positive*, *well we had to lock them up to prevent them from leaving*, *but it goes against the way we treat our residents*.*” (NH1*, *Occupational therapist)*

The shortage of personnel, due to increased sick leaves, also took its toll on the remaining staff as they needed to take over the gaps left by others.

"*We had a lot of staff who were sick*. *We worked understaffed*. *We worked for two months under-staffed without adequate replacements*. *It was really painful and very tiring*. *We were very tired*.*” (NH6*, *Nurse)*

Due to task-shifting, work overload and lack of protective measures and materials, the staff experienced a loss of human contact with their residents. Their normal ways of providing any quality of life care were suspended as residents were sometimes locked in their rooms, and staff were unable to show any affection for their residents. The masks created even more distance, as many residents did not recognize the staff anymore or could no longer engage in a conversation due to hearing problems.

### Emotional effects on staff

The events and changes occurring throughout the pandemic emotionally impacted NH staff and contributed to negative feelings but also some positive attitudes.

Stress, fear and fatigue resulted from the shortage of PPE, the need to work in a totally different way, and being overworked.

“*Sometimes I would undress in the hallway and close the door*. *The children were there and I would go straight up to wash myself because I was also afraid that if I caught the virus in the nursing home I could take it to my home too*, *so it was a double stress*. *When we enter the nursing home we had stress to come home and when we came home there was still stress [*…*] I didn’t even want to cuddle with the children anymore and it was hard*, *especially for my little one*, *he always came*..*” (NH5*, *Nurse-aid)*

Frustration and anger were expressed for the non-recognition of their situation and abandonment by the authorities. Other emotions were great sadness for the loss of many residents who died due to Covid-19, the feeling of powerlessness of not having control over the situation, and guilt for perhaps bringing the virus inside the NH. In addition, some NH staff faced stigma from the surrounding community which caused even more despair, despondency and discouragement.

“*As soon as you say you work in a NH*, *people get scared*, *and even the worst thing is that when you go to the supermarket*, *all the clients*… *they literally run away from you*. *I didn’t dare to go there dressed like that [in uniform] anymore*.*” (NH8*, *Support-staff)*

As staff were and remained very committed to their jobs, priority was given to the wellbeing and protection of residents first, putting their own needs and emotions in the background. *“We have thought more about protecting our residents than we have about ourselves*. *We protected*, *we overprotected*, *our lives were in the background*, *it was the residents first*.*” (NH1*, *Nurse-aid)*

On a positive note, there was a great sense of unity. Staff from all eight nursing homes expressed that peer support and team spirit helped them to overcome difficulties.

“*It really helped that we could talk to each other*, *among colleagues*, *because we all were living and working in the same situation*. *People from outside don’t always understand what it’s like…But we had each other*.*” (NH8*, *Nurse)*

### Needs of residents and staff

Analyses of the interviews revealed five major needs of residents during containment due to Covid-19: social contact, freedom, activities, communication and autonomy. Residents described how they tried to meet these needs, and what helped them pass these difficult months.

“*What was missing the most was being able to talk to people*, *being able to communicate*, *exchange ideas and all that*, *we have to make sure that we can continue this*. *It’s really important for me anyway*, *and for a lot of people I think*, *there are a lot of people who need that*, *human contact*, *because we’re all alone*.*” (NH5*, *96 years*, *male resident)*

The importance and need of social contact was, without exception, confirmed by all interviewed residents and staff members.

“*Among colleagues it was said that contact with the family was capital*. *In the future we must really think about this because contact with the family is very important*.*” (NH6*, *Nurse)*

This need was why the paramedical staff expressed the urgent need to continue their own jobs, especially those in relation to social liaison, as an occupational therapist emphasized: *“In the end*, *what is our role in this nursing home*, *it’s the social link*, *it’s fighting against loneliness and all that*. *At a certain point we have to refocus*, *to continue the work of accompanying the elderly in the nursing home*.*” (NH3*, *Occupational therapist)*

Several residents were allowed to have contact with other residents, organized as ‘bubbles’ within the NH. Others were able to continue to contact with their family via their window, balcony, in a garden, or by telephone. Residents described all sorts of social contact and connectedness with the outside world as helpful.

Another coping mechanism for several residents was receiving more information about the pandemic and the NH’s response, giving them a sense of understanding. The extra information, new perspectives, and possibilities to go outside (in the garden), made them feel useful and included again.

There was one, small in size, NH in particular where a lot of these good practices took place, and the emotional impact was lower, both among staff and residents. Due to its small size and its existing family atmosphere between staff and residents, it was already normal practice to involve residents in decision-making processes. This did not change during the first wave in which residents were involved in the policy design of room isolation, resulting in the creation of special bubbles among residents who chose to stay together as opposed to solitary isolation, while being well aware of the risk of infection. When there was not enough PPE material, this was openly discussed with the residents; the result was that some residents got involved in the creation of these.

“There are residents who know how to sew, one’s who knows how to cut, and those who have a [sewing] machine. We asked who knows how to sew, who wants to do it, and they made some masks.” (NH8, Nurse-aid)

But in all seven other NHs, where communication platforms were not (adequately) in place, residents strongly requested for more freedom. They wanted to be able to “*go out*”, “*go for a walk*”, they wanted *“air”*, *“a change of situation”*, they wanted *“to live”*.

“*My freedom and to lead my life*, *which I have always done*. *I need it*, *as long as I have my brain (she laughs)*. *I need to continue to lead my life [*…*] I can’t stand people interfering with what I want to do or not do*.*” (NH7*, *83 years*, *female resident)*

To fight against their boredom and loneliness, residents welcomed all sorts of distraction. Quite a few distractions were not too difficult to implement, as one resident emphasized: *“A little music*… *with music it will be better*, *it gives a good atmosphere*, *it distracts*, *it softens*” *(NH7*, *82 years*, *female resident)*

Residents were thankful for little individualized treats from the NH staff. Although staff were very limited in time and often had no time to talk, residents’ stories showed that, for them, an attentive and respectful attitude of the professional caregiver was far more important than the actual time they spent with them.

“*The director is intelligent*, *understanding*, *discreet*, *everything we want*. *The staff is always super friendly*, *we have a very good atmosphere here*, *a family atmosphere*.*” (NH8*, *94 years*, *female resident)*

Staff expressed a dire need of support in dealing with all the stress and emotions, for themselves as well as for the residents. They believed that family visits could relieve both staff and residents from a lot of distress.

“*Either it’s a psychological help in the nursing home for example or once a week and talking like this*, *it feels good*, *it helps to get out the emotions that were left inside so I think it could be a good thing*.*” (NH4*, *Support staff)*

Finding adequate communication channels was a challenge for everybody, leaving the staff feeling incompetent and lost, and residents excluded. Some residents reported the feeling that staff were not informed, or were afraid to inform them, to protect them from bad news.

“*I understand that they didn’t say it so as not to create anxiety*, *but it did*, *it did create anxiety because we didn’t know what it was about*, *we felt like hostages*, *locked up in the same cauldron”*. *(NH7*, *84 years*, *female resident)*

Staff expressed the need for support on how to communicate bad news, in particular, while more in general it was felt that support was needed on how to have a better and more coherent organization in case of a next outbreak. *“External support is needed to organize the inside*.*” (NH5*, *Support Staff)*

## Discussion

In this study, we sought to describe which psychosocial and mental health needs were identified and experienced by residents of NHs during the first wave and lockdown phase of the Covid-19 pandemic in Belgium and how NH staff perceived their preparedness to address those needs. Our findings showed that the lockdown measures strongly affected the residents’ life and well-being. The loss of freedom, social life, activities and autonomy had a massive impact on residents’ mental well-being as it resulted in a huge perceived decrease of their meaning and quality of life. Meanwhile, NH staff, who observed these negative effects, were abruptly confronted with professional and ethical dilemmas. They were continuously challenged with organizational changes, lacking or conflicting information and protocols and a lack of PPE. This left them feeling that the desperately needed good quality of care for residents was slipping away beyond their will.

The Covid-19 related restrictions challenged three Basic Psychological Needs [23, 24]: “autonomy (one’s ability to regulate own actions, having a sense of freedom and choice), competence (one’s efficiency in interacting with the environment, the feeling of control and mastery) and relatedness (one’s ability to search for and develop connections in interpersonal relationships)”. These universal needs are essential to human functioning and psychological well-being [25, 26]. According to the resident’s accounts, except from one NH, by not being informed or included in pandemic preparedness, their autonomy was abruptly taken away. Their need for competence was not met as residents felt useless and ‘abandoned’, and cut off from society, while their ability to relate to others was hampered if not totally obstructed. We, therefore, conclude that the experiences of residents during Covid-19’s first wave can be characterized as a deprivation of the residents’ Basic Psychological Needs, resulting in poorer mental well-being. These findings are complemented by a recent study of Cantarero et al. which showed that deteriorating mental well-being during the pandemic stems from frustration of these Basic Psychological Needs [27].

In addition to loss of Basic Psychological Needs, other research has indicated that loneliness and social isolation have negative consequences for residents’ overall health and well-being [9, 10, 28]. Besides the ban on family visits, health professionals such as physicians, psychologists, physiotherapists, and service providers such as hairdressers, were no longer able to enter NHs. Except from one NH, group-based and social activities were no longer organized, and meals that were previously shared together, were now consumed alone in residents’ rooms. All these safety measures increased residents’ social isolation and loneliness. Similar observations have been described in other countries such as Germany [29]. It is, therefore, not surprising that the impoverished environment and the lack of regular social, cognitive and physical stimulation had an enormous impact on residents and their well-being. Residents described feelings of depression, hopelessness, uselessness, and sadness; some even expressed a wish to die. Those who felt infantilized or deprived of information became frustrated or even angry, resulting in resistance to the lockdown measures.

Meanwhile, every day, NH staff witnessed the detrimental effects of the lockdown measures on their residents and faced the dilemma of choosing infection prevention versus allowing social contact for residents. The staff have close relationships with their residents, looking after them since months and years. During the first wave lockdown, while trying to keep ‘their’ residents safe, at the same time they saw them lose their appetite for food and life. It was a silent and hidden loss, but all the more distressing. Considering this, it was not surprising that many health care professionals suffered from stress due to ethical dilemmas, usually referred to as “moral distress” [30, 31]. Priorities changed during the pandemic, from a resident-centered approach to a collective-protective plan which led to unintended consequences. As a result, NH staff were caring for residents in ways that would not have been optimal pre-pandemic [32, 33]. In particular, staff felt unprepared for the psychosocial and mental health challenges posed by the pandemic. They were constantly adapting to the medical and infection control situation but lacked guidelines, information and support to address mental health needs. They reported feeling “abandoned”.

Our findings clearly show that the consequences of Covid-19 in NHs go beyond physical health, as emotional distress, anxiety, depression, public stigma and sleep disturbances reflected problems with psychological health, not only among residents but also for their caregivers. The emotional burden on the staff was and still is high [34], affecting their attention, understanding and decision-making capacity and having long term consequences on their general well-being. Some stressors could have been prevented by timely implementation of PPE, safety guidelines and psychosocial support, as confirmed by a recent study on the mental health impact of Covid-19 on NH staff in Poland [35]. This is in line with our findings, as NH staff identified the lack of PPE, lack of safety guidelines and coherent organization and lack of external support as the main stressors. Several mental health initiatives were launched in Belgium, both online or by telephone. Unfortunately, these rely on the initiative of the health care worker to seek help, and for that reason they were not used by the staff. A good practice of Iriscare, the public health institute of Brussels, was to proactively call all nursing homes and offer psychosocial support in the nursing homes. However, even though Iriscare was often able to offer good help, due to different sorts of barriers this psychosocial support could not always reach the staff in distress.

There are some limitations to this study that should be taken into account. We only investigated the experiences of residents who did not exhibit reduced cognitive capacities; therefore, the experiences of elderly with dementia or other cognitive problems, who are even more vulnerable, were not included. The study period also overlapped the time from full lockdown to partial lockdown during which some participants were receiving limited visits or were expecting them soon. This may have biased their responses and limited our time for conducting data collection as it was only planned for the first lockdown-phase.

The findings of this study during the first wave of Covid-19 suggest several urgent points of attention for health authorities to create policies for providing safe and humane care to the vulnerable residents for next waves. The practice of social distancing and physical separation remains important to keep residents in NHs safe, but the time has come to re-think policies. This study’s findings emphasize the urgent need to use a more comprehensive approach, and while protecting the residents by implementing IPC measures, preventive strategies to reduce the mental health impact of these imposed measures need to be prioritized. Good practices such as inclusion of residents in planning, transparent communication, creative thinking and other measures where one tries to re-take control, can facilitate coping strategies to better deal with the difficult situation, and hence reduce its emotional impact. In the one small-sized NH where these good practices were present, communication had always been of a natural participatory nature. The discussion on small-sized institutions versus, often due to its size, bureaucratic big institutions goes beyond the objective of this paper.

The indispensable support of family members, benefiting both residents and staff members, needs to be incorporated into care under controlled IPC measures. Verbeek et al. showed that it is possible to allow family visits with good compliance to local IPC guidelines [36]. The concept of “Essential Family Caregivers” [37] should be considered. To improve residents’ autonomy, the Tubbe model is strongly recommended [38]. It was inspired by the vision of the Danish educational expert Tyra Frank and following her motto “*As long as one is alive*, *one should live*”. The Tubbe organizational model for care homes is based on a genuine participative management process which includes residents at all levels in the planning and running of their own living conditions. Psychosocial support and mental health services should be made more easily available for both residents and staff [39]. Despite the pandemic, life in NHs has to continue, offering meaningful activities, including residents in decision-making processes and allowing them more control over their own living situation. It is essential that residents feel useful and included in our society.

## Conclusion

This study made it clear that to prepare NHs for Covid-19, a holistic approach is needed recognizing that psychological needs are as important as physical health needs. Protecting the elderly during Covid-19 means not only shielding them from the virus, but also taking into account their autonomy and individual understanding of quality of life. Psychosocial factors must be considered as potential source of suffering, and evaluated through specific diagnostic tools. More creative and preventive interventions need to be designed to support and enhance residents’ resources and coping strategies, and accordingly their psychosocial and mental well-being during the pandemic. In turn, better planning and support for staff will reduce their stress in having to deal with the stress of choosing physical protection versus psychological care for residents in their care.
