# Correction: How to bring residents’ psychosocial well-being to the heart of the fight against Covid-19 in Belgian nursing homes—A qualitative study

**DOI:** 10.1371/journal.pone.0299576

**Published:** 2024-02-22

**Authors:** Sanne Kaelen, Wilma van den Boogaard, Umberto Pellecchia, Sofie Spiers, Caroline De Cramer, Gwennin Demaegd, Edouard Fouqueray, Rafael Van den Bergh, Stephanie Goublomme, Tom Decroo, Muriel Quinet, Elke Van Hoof, Bertrand Draguez

There are errors in the Funding and Competing Interests statements. The correct statements are:

**Funding**: Médecins Sans Frontières (MSF) provided support in the form of salaries for SK, WvdB, UP, SS, CdC, GD, FE, RvdB, SG, and BD. The specific roles of these authors are articulated in the ‘author contributions’ section. MSF programmatic funding covered all costs associated with the study. MSF was involved in the study design, data collection and analysis, decision to publish, and preparation of the manuscript. No other forms of support were provided specifically for the research. No grants were provided. Research conducted by MSF employees is done so within the scope of their job specification.

**Competing Interests**: The authors have read the journal’s policy and have the following competing interests to declare: MSF provided support for this study in the form of salaries for SK, WvdB, UP, SS, CdC, GD, FE, RvdB, SG, and BD. This does not alter our adherence to *PLOS ONE* policies on sharing data and materials. There are no patents, products in development or marketed products associated with this research to declare.
